# Remineralization Effect of Bioactive Glass With and Without Fluoride and Casein Phosphopeptide-Amorphous Calcium Phosphate (CPP-ACP) on Artificial Dentine Caries: An In Vitro Study

**DOI:** 10.7759/cureus.70801

**Published:** 2024-10-03

**Authors:** Kinza Manzoor, Sadia Manzoor, Zunaira Qazi, Sundas Ghaus, Mehvish Saleem, Muhammad Kashif

**Affiliations:** 1 Dental Biomaterials, Islamic International Dental College, Islamabad, PAK; 2 Dental Biomaterials, Bakhtawar Amin Medical and Dental College, Multan, PAK; 3 Oral Pathology, Bakhtawar Amin Medical and Dental College, Multan, PAK

**Keywords:** artificial caries, bioactive glass, cpp-acp, dentine, fluoride, remineralization

## Abstract

Background/objectives

Dentine hypersensitivity (DH) is a common dental condition marked by transient, sharp pain arising from dentinal exposure. Bioactive materials are capable of remineralization. This study aims to explore the remineralization effect of bioactive glass (BAG) with and without fluoride and casein phosphopeptide-amorphous calcium phosphate (CPP-ACP), using dentine discs as the test substrate.

Materials and methods

In this in vitro experimental study, 28 dentine discs were prepared from premolar teeth. Artificial caries were induced by subjecting the dentine discs to demineralization in acid for 72 hours. After demineralization, the discs were treated with various remineralizing dentifrices to evaluate their effects. The dentine discs (n=28) were divided into four groups: group 1 = BioMin (BioMin Technologies, London, United Kingdom), group 2 = NovaMin/Sensodyne Repair (GlaxoSmithKline plc, London, England, United Kingdom), group 3 = Recaldent (CPP-ACP; GC Orthodontics Inc., Alsip, Illinois, United States), and group 4 = deionized water (control group). All discs underwent a 28-day remineralization process using the respective dentifrices assigned to each group. The microhardness of the discs was measured using Vickers microhardness testing at three stages: baseline, post-demineralization, and post-remineralization.

Results

There was no significant difference in terms of microhardness between groups both at the baseline (F (3, 24) = 1.079, p = 0.995) and after the demineralization process (F (3, 24) = 1.310, p = 0.294). However, a significant difference was identified between the groups after the remineralization process (F (3, 24) = 34.008, p = 0.001). Additionally, the least significant difference (LSD) multiple comparison test was performed. There were significant differences identified in the remineralization condition, with group 1 having the highest values, followed by group 2, group 3, and group 4.

Conclusion

The results indicate that the remineralization effect on artificially induced dentine caries was more pronounced with bioactive glass containing fluoride compared to bioactive glass without fluoride and CPP-ACP.

## Introduction

Dentine hypersensitivity (DH) is a neurological reaction to external stimuli in the oral environment, which typically lasts for a few seconds to a couple of minutes. It occurs when the dentine tubules become exposed due to factors such as gum recession or damage to the protective enamel, allowing stimuli to affect the nerves in the pulp and trigger a neurological response. This phenomenon is explained by hydrodynamic theory, which was described by Brannstrom in 1963 [1]. Various stimuli, including pressure, temperature, and osmotic changes, can influence the fluid flow within the dentine tubules, leading to nerve distortion and the resulting hypersensitivity. Additionally, the consumption of sugary, salty, and acidic foods, which are considered high-osmotic stimuli, can also contribute to alterations in the fluid dynamics within the dentine tubules [2]. This loss of enamel can result from factors such as tooth wear, gum recession, poor dental hygiene, excessive and aggressive brushing, or acid erosion. However, acidic conditions in the oral environment can rapidly dissolve this smear layer, exposing the tubules of dentine. Under in vitro conditions, this smear layer can be dissolved within as little as a three-minute duration. Dental dentifrices, which include toothpaste, can also contribute to the removal of this smear layer due to the presence of abrasive particles designed to eliminate plaque and stains. DH is one of the most prevalent oral conditions with an incidence of up to 30% and this condition is more prevalent among individuals aged 30-40 years [3,4].

The 45S5 bioactive glass (BAG) (introduced in the early 1970s), falls under the category of glasses within the Na_2_O-CaO-SiO_2_-P_2_O_5_ system and is notably rich in calcium content [5]. This type of BAG was discovered to have the capacity to form a rapid and robust bond with bone tissue. Furthermore, it has the ability to stimulate bone growth, promoting new bone formation away from the bone-implant interface [6]. Research conducted previously confirmed the formation of a robust bond between dentine and BAG, resulting in the build-up of ions such as calcium and phosphate on the dentine's surface [7]. It was subsequently conjectured that this material possesses the capability to create a protective layer on the dentine surface, offering relief from DH. Calcium sodium phosphosilicate (CaNaO_6_PSi, CSPS) exhibits a rapid ion exchange mechanism, wherein it promptly exchanges sodium ions (Na+) with hydrogen ions (H+) upon contact with saliva or any aqueous medium.

The process involves the rupture of silicon with oxygen bonds, leading to the formation of silica with Si-OH groups. This breakage in the silica network results in the release of calcium and phosphate ions. These ions subsequently undergo precipitation, forming a calcium phosphate (Ca-P_2_O_5_) layer on the tooth's surface [8]. Furthermore, this layer binds to collagen fibers, contributing to the overall mechanism of action. It is also reported that replacing CaO with CaF2 in the glass composition would indeed reduce reactivity due to the alteration in the glass network structure [9]. This substitution decreases the number of non-bridging oxygen (NBO) sites in the glass network, resulting in a more crosslinked structure. This increased crosslinking prevents the glass from undergoing condensation and repolymerization reactions that are essential for bioactivity. Consequently, the glass becomes less reactive in biological environments [10].

This study focused on the remineralization of dentine using Vickers microhardness to compare the mineral density attained after treatment with BAG with fluoride and BAG without fluoride and CPP-ACP. There is a gap in the literature regarding the level of protection by the layers formed with these pastes containing BAG and CPP-ACP. Artificial saliva used in this study was a true representation of the oral environment as the dentine comprised 22 percent water in volume in vivo. The null hypothesis (H0) was that there was no difference in the efficacy of the examined dentifrices in occluding dentinal tubules.

## Materials and methods

Research protocol

**Figure 1:**
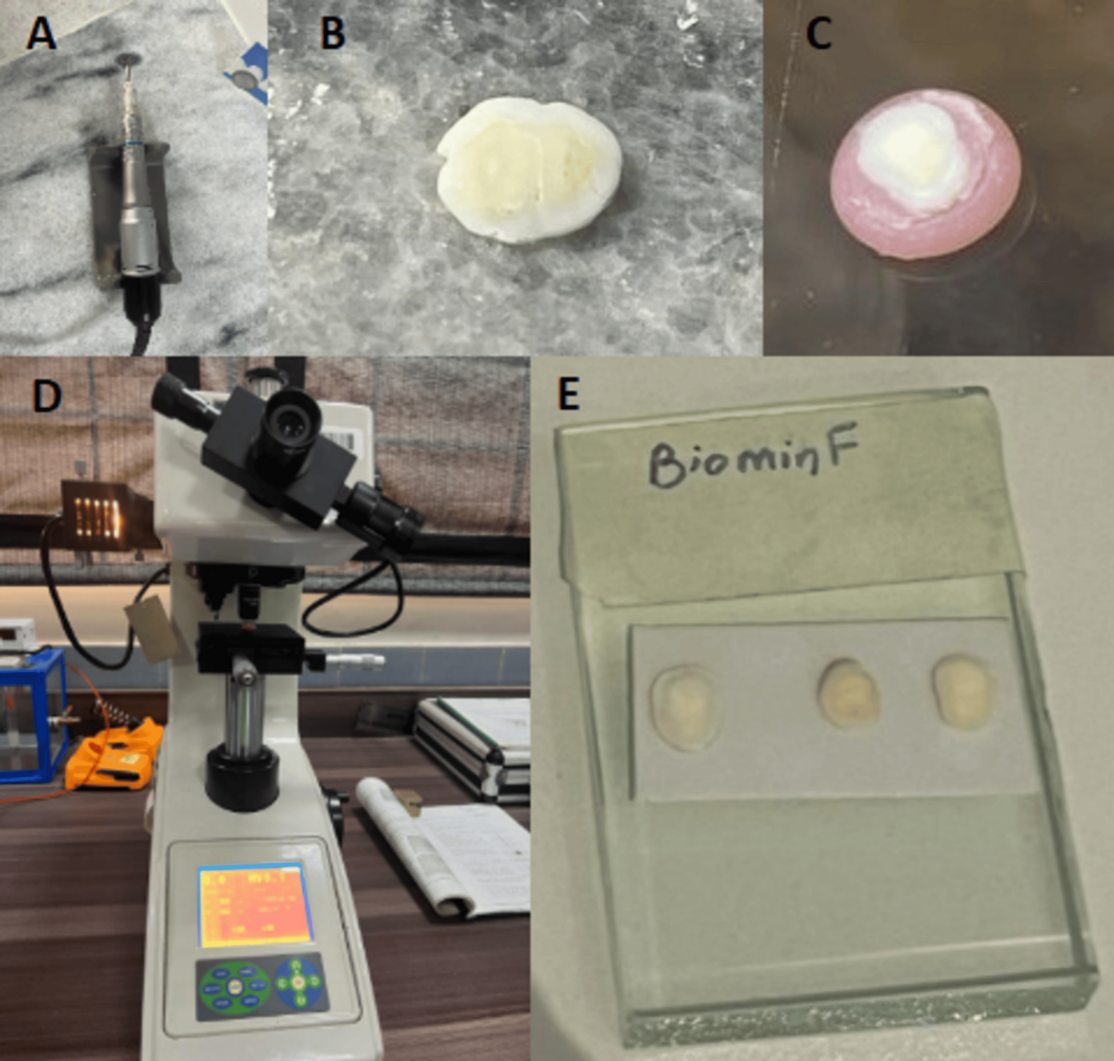
(A) Slow-speed micromotor used with diamond disc for dentine disc preparation. (B) Prepared dentine disc. (C) Dentine disc mounted on an acrylic resin block for Vickers micro-indentation. (D) Dentine disc placed on a Vickers indentor for microhardness testing. (E) Dentine disc placed on a glass slab with double-sided tape for paste application.

The study received ethical approval from the Ethics Committee of the Islamic International Dental College, Riphah University in Islamabad, Pakistan (Ref. No. IIDC/IRC/2022/012/007). This study was conducted over a duration of seven months from January 2023 to July 2023 after the approval of the synopsis. All participants in the study received dental care at the same institution and provided written informed consent. Parents or legal guardians of teenagers under the age of 16 were asked to provide written consent for their participation in the experiment. The sample size for each group was calculated using the OpenEpi sample size calculator (Dean AG, Sullivan KM, Soe MM. OpenEpi: Open Source Epidemiologic Statistics for Public Health, Version. www.OpenEpi.com, updated 2013/04/06), with a confidence interval of 95% and a power of 80%. By comparing the mean difference between the groups, the calculated sample size was three in each group, but we have adjusted the sample size up to seven in each group [11]. A total of 28 human premolars were extracted for orthodontic reasons and preserved in deionized water with 0.1% thymol at 4°C. Teeth with cavities, restorations, or crown fractures were excluded from the study. Using a low-speed micromotor, 28 dentine discs, each 1.0 mm thick, were prepared by cutting each tooth crown (mesiodistally) over the cementoenamel junction, and along the tooth's long axis. The occlusal cusp tip was also removed. The coronal side of the dentin surface was rubbed with silicon carbide papers using 600-grit, 800-grit, and 1200-grit, which was followed by washing the discs (Figure 1).

The discs were randomly separated into four groups, each containing seven dentine discs. There were four groups consisting of different commercially available pastes (Table 1).

**Table 1:** Study groups' nomenclatures and compositions BioMin: BioMin Technologies, London, United Kingdom; NovaMin: GlaxoSmithKline plc, London, England, United Kingdom; Tooth Mousse: GC Orthodontics Inc., Alsip, Illinois, United States

Number of group	Name of group	Composition	Commercial name
Group 1	BIO	Bioactive glass (BAG) with fluoride	BioMin
Group 2	NOV	BAG without fluoride	NovaMin
Group 3	CPP	Casein phosphopeptide-amorphous calcium phosphate	Tooth Mousse
Group 4	W	Deionized water	Deionized water

The microhardness of the seven discs of each group was taken at this point as the baseline microhardness of dentine discs. After that the demineralization solution was prepared and all the discs of the four groups were immersed in it in their separate containers for 72 hours.

Demineralization solution

The demineralization solution was composed of (0.05 M) acetic acid containing 2.2 mM calcium chloride dihydrate (CaCl_2_·2H_2_O) and 2.20 mM potassium dihydrogen phosphate (KH_2_PO_4_) and was adjusted to pH 5.0. After taking out all the discs from the demineralization solution, the discs were thoroughly washed with distilled water and the microhardness of the seven discs from each container was measured. This is called the demineralization microhardness [12].

Artificial saliva (AS)

After demineralization, these seven dentine discs were placed in four separate labeled containers having AS. AS was composed as explained by Fusyama et al. [13]. The pH of freshly prepared AS was more acidic, which was adjusted to 7.0 by the addition of 1 M sodium hydroxide [14]. The AS solution was kept refrigerated at around 5°C and was used within seven days after manufacturing.

Application of remineralization agents

For the four different groups, seven specimens (n = seven) were placed in separate containers containing AS. These samples were moistened with deionized water, and an automatic toothbrush, Nero Pro Toothbrush (Nero, Leicester, United Kingdom), was employed to apply the respective products onto the dentine discs. The specimens underwent the remineralization process twice a day (morning and evening) for 28 days [11]. For brushing specimens from group 1 (BioMin; BioMin Technologies, London, United Kingdom), a 20 mL slurry of toothpaste was mixed with AS in a ratio of 1:3. For group 2 (Sensodyne Repair and Protect Deep Repair, powered by NovaMin; GlaxoSmithKline plc, London, England, United Kingdom), a 20 mL toothpaste slurry was used, in a ratio of 1:3 paste to saliva. A 20 mL toothpaste slurry mixed with AS in a ratio of 1:3 was used for group 3 (CPP-ACP group), and for group 4 (water group), 20 mL of deionized water was used. All the dentine discs were removed from their containers, brushed with pastes for two minutes, and gently rinsed with deionized water. All teeth were again soaked back in artificial saliva at 37°C. Microhardness testing was done after four weeks.

**Figure 2:**
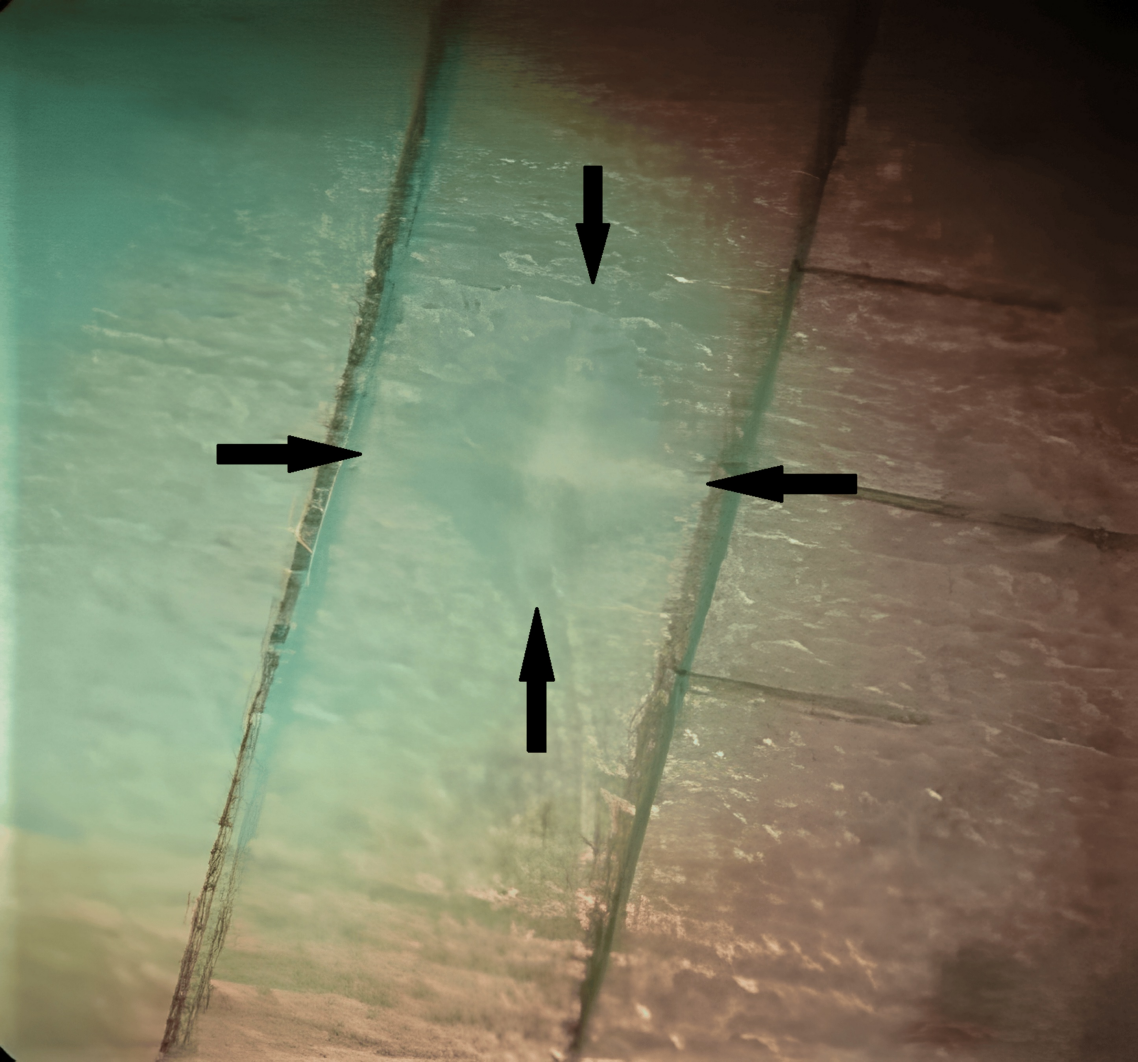
Diamond indent with a Vickers microhardness tester on a dentine disc. The Vickers number could be determined by the diamond depression, corresponding to the anticipated surface apices of the diagonals.

Each group's seven discs were chosen at random to assess the microhardness at three different times: Vickers hardness at baseline (VHNba), Vicker's hardness after demineralization (VHNde), and Vickers hardness after remineralization (VHNre) paste application. A Vickers indenter was used to measure each disc's microhardness value on a microhardness tester (Wolpert Wilson Instruments 402 MVD, Illinois Tool Works, Glenview, Illinois, United States). Three similar but different areas were indented using a Vickers diamond indenter. Measuring the long and short diagonals of the indentations in dentin was considered sufficient for indentations made with a load of 0.98 N over 15 seconds, as this caused minimal surface damage (Figure 2). The Vickers number could be determined by the diamond depression, corresponding to the anticipated surface apices of the diagonals. The ΔVHN is the difference between the microhardness after remineralization and microhardness after demineralization (ΔVHN = VHNre - VHNde) [11].

The microhardness was calculated using the following formula, and the average of the three measurements was calculated and reported as the microhardness value in Vickers hardness units: Hv=1.8544×Pd2, where Hv is the Vickers hardness number in kg/mm2, P is the indenter load in kg, and d is the diagonal length of the impression in mm.

Statistical analysis

The Shapiro-Wilk test was employed to determine the data's normality. A one-way analysis of variance (ANOVA) was used when p<0.05 to compare the Vickers of baseline, demineralization, and remineralization among the four groups. In addition, the LSD multiple comparison test was performed. IBM SPSS Statistics for Windows, Version 26, (Released 2019; IBM Corp., Armonk, New York, United States) was used for all statistical analyses. For all analyses, the significance level was set at ≤0.05.

## Results

Microhardness baseline analysis

There were seven discs in each group. Three values were averaged to make the final value of each disc. The Vickers values for all groups are presented in Table 2 under three conditions: baseline, demineralization, and remineralization. It can be noticed that the deionized water group has the lowest, while the BioMin group has the highest mean Vickers microhardness values.

**Table 2:** Descriptive statistics of Vickers hardness (kgf/mm2) at baseline, demineralization, and remineralization (mean±SD) Bio: BioMin; Nov: NovaMin; CPP: CPP-ACP, casein phosphopeptide-amorphous calcium phosphate; W: deionized water BioMin: BioMin Technologies, London, United Kingdom; NovaMin: GlaxoSmithKline plc, London, England, United Kingdom; Tooth Mousse: GC Orthodontics Inc., Alsip, Illinois, United States

Group	Baseline (mean±SD)	Demineralization (mean±SD)	Remineralization (mean±SD)
Bio	62.6857±3.3289	23.9286±7.0401	60.0571±5.9796
Nov	61.7571±4.2747	27.6857±2.1177	47.5286±8.0795
CPP	62.0043±8.9632	27.4571±1.512	40.5286±6.9801
W	62.1571±8.9796	26.5571±2.6639	27.3286±1.9956

The ANOVA test was used to investigate the differences in Vickers microhardness values across groups under the baseline, demineralization, and remineralization conditions (Table 3). There was no significant difference between groups for the baseline condition (F (3, 24) = 1.079, p = 0.995). Similarly, there was no significant difference between groups for the demineralization condition (F (3, 24) = 1.310, p = 0.294). However, a significant difference was identified between the groups for the remineralization condition (F (3, 24) = 34.008, p = 0.001). To ascertain the exact group differences, additional post-hoc testing would be required. There were no significant changes in Vickers values between the groups at the baseline and demineralization conditions. At the remineralization condition, however, a significant difference was noticed between the groups.

**Table 3:** Analysis of variance (ANOVA) across groups under the baseline, demineralization, and remineralization conditions.

Vickers microhardness		Sum of squares	Df	Mean square	F	Significance
At baseline	Between groups	3.238	3	1.079	.023	.995
	Within groups	1141.954	24	47.581		
	Total	1145.192	27			
On demineralization	Between groups	62.321	3	20.774	1.310	.294
	Within groups	380.577	24	15.857		
	Total	442.899	27			
On remineralization	Between groups	3921.347	3	1307.116	34.008	.000
	Within groups	922.440	24	38.435		
	Total	4843.787	27			

Table 4 shows the results from multiple comparisons of Vickers values between different groups using the LSD method under the baseline, demineralization, and remineralization conditions. In the baseline and demineralization phases, there were no significant differences in Vickers values observed among the groups. However, during the remineralization phase, distinct variations emerged. Specifically, group 1 exhibited significantly higher Vickers values when compared to group 2, group 3, and group 4. Additionally, significant differences were noted when comparing individual groups. The Nov (NovaMin) group had significantly higher Vickers values than the Bio (BioMin), CPP (CPP-ACP), and W (deionized water) groups. Similarly, the CPP group demonstrated significantly higher Vickers values compared to the Bio, Nov, and W groups, while the W group showed significantly higher values when compared to the Bio, Nov, and CPP groups. These findings highlighted notable disparities in the effects on Vickers values during the remineralization phase across the various treatment groups. Overall, the findings indicate that there were no significant variations in Vickers values across groups in the baseline and demineralization conditions. However, there were significant differences identified in the remineralization condition, with the Bio group generally having the highest values, followed by the CPP group and then the W group.

**Table 4:** Multiple comparisons of least significant difference (LSD) Vickers values (kgf/mm2) between different groups under the baseline, demineralization, and remineralization conditions *The mean difference is significant at the 0.05 level Bio: BioMin; Nov: NovaMin; CPP: CPP-ACP, casein phosphopeptide-amorphous calcium phosphate; W: deionized water BioMin: BioMin Technologies, London, United Kingdom; NovaMin: GlaxoSmithKline plc, London, England, United Kingdom; Tooth Mousse: GC Orthodontics Inc., Alsip, Illinois, United States

Dependent variable	(I) Group	(J) Group	Mean difference (I-J)	Standard error	Significance	95% confidence interval	
						Lower bound	Upper bound
Vickers baseline	Bio	Nov	.92857	3.68710	.803	-6.6812	8.5384
		CPP	.68143	3.68710	.855	-6.9284	8.2912
		W	.52857	3.68710	.887	-7.0812	8.1384
	Nov	Bio	-.92857	3.68710	.803	-8.5384	6.6812
		CPP	-.24714	3.68710	.947	-7.8569	7.3627
		W	-.40000	3.68710	.915	-8.0098	7.2098
	CPP	Bio	-.68143	3.68710	.855	-8.2912	6.9284
		Nov	.24714	3.68710	.947	-7.3627	7.8569
		W	-.15286	3.68710	.967	-7.7627	7.4569
	W	Bio	-.52857	3.68710	.887	-8.1384	7.0812
		Nov	.40000	3.68710	.915	-7.2098	8.0098
		CPP	.15286	3.68710	.967	-7.4569	7.7627
Vickers demineralization	Bio	Nov	-3.75714	2.12854	.090	-8.1502	.6359
		CPP	-3.52857	2.12854	.110	-7.9217	.8645
		W	-2.62857	2.12854	.229	-7.0217	1.7645
	Nov	Bio	3.75714	2.12854	.090	-.6359	8.1502
		CPP	.22857	2.12854	.915	-4.1645	4.6217
		W	1.12857	2.12854	.601	-3.2645	5.5217
	CPP	Bio	3.52857	2.12854	.110	-.8645	7.9217
		Nov	-.22857	2.12854	.915	-4.6217	4.1645
		W	.90000	2.12854	.676	-3.4931	5.2931
	W	Bio	2.62857	2.12854	.229	-1.7645	7.0217
		Nov	-1.12857	2.12854	.601	-5.5217	3.2645
		CPP	-.90000	2.12854	.676	-5.2931	3.4931
Vickers remineralization	Bio	Nov	12.52857^*^	3.31382	.001	5.6892	19.3680
		CPP	19.52857^*^	3.31382	.000	12.6892	26.3680
		W	32.72857^*^	3.31382	.000	25.8892	39.5680
	Nov	Bio	-12.52857^*^	3.31382	.001	-19.3680	-5.6892
		CPP	7.00000^*^	3.31382	.045	.1606	13.8394
		W	20.20000^*^	3.31382	.000	13.3606	27.0394
	CPP	Bio	-19.52857^*^	3.31382	.000	-26.3680	-12.6892
		Nov	-7.00000^*^	3.31382	.045	-13.8394	-.1606
		W	13.20000^*^	3.31382	.001	6.3606	20.0394
	W	Bio	-32.72857^*^	3.31382	.000	-39.5680	-25.8892
		Nov	-20.20000^*^	3.31382	.000	-27.0394	-13.3606
		CPP	-13.20000^*^	3.31382	.001	-20.0394	-6.3606

## Discussion

This research delved into examining how the combination of BioMin and NovaMin affects the remineralization of artificial dentine caries. It offers valuable insights into the alterations in the microstructure of dentine caries following the application of BAG. As per the study's findings, the initial hypothesis was disproven. BAG with fluoride and BAG without fluoride demonstrated a promising capacity for remineralization in artificial dentine caries, leading to an increase in microhardness by forming a mineralized layer on dentin and assessing the tubules occlusion by comparing their effectiveness to standard CPP-ACP and water. Studies have indicated that fluoride's capacity to promote dentin remineralization is constrained when there are insufficient residual crystals within the lesion. While fluoride is highly effective in the remineralization of enamel, its impact on DH is somewhat limited [15]. Two approaches have been emphasized to achieve remineralization of dentine caries: one involves applying nucleation templates onto demineralized dentin, while the other focuses on establishing a localized environment with elevated concentrations of calcium and phosphorus [16].

Since bone and dentine share similar chemical compositions, it is reasonable to anticipate that a material capable of forming a robust bond with one will likely exhibit a similar bonding capability with the other [17]. In a previous study, a very fine BAG powder with a grain size of less than 90 nm was used. Tiny particles with a small size enable easier penetration into dentine caries and offer a larger surface area for chemical reactions [18]. ten Cate concluded that calcium could be a limiting factor in the mineralization of carious dentine [19]. The inclusion of fluoride in BAG composition facilitates a gradual and continuous release of fluoride, allowing for the potential formation of long-lasting fluorapatite (FAP) rather than fluorite. This sustained release can intensify the protective effects of fluoride on dental tissues [20]. A study conducted by Farooq et al. examining the addition of fluoride to a BAG composition found that fluoride had the effect of softening the glass, making it more bioactive. This increased bioactivity could potentially enhance the glass's interaction with dental tissues and its therapeutic benefits [20]. Examining hardness is an indirect approach for monitoring alterations in the mineralization of dental tissue, and numerous studies focusing on dentine increase in microhardness have been documented [21,22]. These findings are consistent with another study that examined the potential for enamel remineralization with NovaMin, where it was observed that both calcium and phosphate content increased significantly [23].

The strong bonding capacity of BAG to tooth structure could be a significant factor contributing to its enhanced remineralization effect. Based on the findings of this in vitro study, we conclude that BAG encourages the remineralization of artificial dentin. BAG holds promise as a potential alternative to fluoride and CPP-ACP in the treatment of dental caries [11]. Nanoindentation measurements in the current study showed that the layers formed as a result of the use of these toothpastes are harder and stiffer than the control dentine. The hardness for dentine (0.55±0.05 GPa) was within the range for intertubular dentine [24-26]. However, some authors have reported higher hardness values for dentine. For example, the nanoindentation data by Yassen et al. revealed that dentine had a hardness of 0.89 GPa [27]. Another nanoindentation experiment on human dentine from a third molar by Habelitz et al. showed that dentine had a modulus of 23.7 GPa; this finding was in agreement with the finding by Zheng et al. that revealed dry dentine had a reduced modulus of 24 GPa [27-29].

The control dentine discs were kept in AS and were tested while fully immersed in deionized water. Therefore they were fully hydrated, which can explain the lower values compared to these studies. Our values for microhardness are also between the values quoted in the literature, and it can be due to the dentine discs being stored in AS and not being dry. Aguiar et al. investigated the hardness of dentine after treatment with toothpaste containing CSPS and AG by using microhardness measurements with a load of 0.5 N. Their hardness value for control dentine was 0.54 GPa. The hardness increased to 0.64 GPa and 0.67 GPa after treatment with CSPS and CPP-ACP, respectively, which weren’t statistically significant [30]. As well as shielding the tubules from stimuli, this layer needs to be acid- and abrasion-resistant to be able to protect the dentine in the harsh oral environment. After the acid challenge, the results showed that the treated samples had a significantly lower hardness as compared to the original dentine. However, after remineralization, we saw that the microhardness increased differently for different groups, and this was verified by the microhardness test. In the current study, we found out that group 1 (BioMin) has shown the highest remineralization, which was proven with the highest values of microhardness as compared to groups 2,3, and 4.

The remineralization in vitro might differ significantly from a dynamic biological system that often takes place in the mouth cavity in vivo. Because of the inherent limitations of in vitro investigations, caution must be taken when making direct extrapolations to clinical circumstances. Well-controlled clinical trials in real-life dynamic in vivo conditions should be conducted for better insights and benefits. Further investigation and analysis are necessary before application to the broader population.

## Conclusions

Overall, the findings demonstrate no significant differences in Vickers hardness values across the groups under baseline and demineralization conditions. However, significant variations were observed during the remineralization phase, with the BAG group showing the highest values, followed by the CPP group and the W group. The superior tubule occlusion and enhanced dentine mineralization observed in the BioMin group suggest its efficacy in providing greater protection against dentine hypersensitivity. Compared to NovaMin and CPP-ACP, BioMin emerged as the most effective agent for hypersensitivity treatment through enhanced remineralization.
